# Krankenhaus 2030 – Was sich ändern muss

**DOI:** 10.1007/s00772-023-00986-6

**Published:** 2023-03-10

**Authors:** Rainer Minz, Damian Grüttner, Marlis von Heusinger-Lender

**Affiliations:** 1 Aufsichtsrat, Universitätsklinikum Köln (AöR), Köln, Deutschland; 2 Vorstand, Universitätsklinikum Köln (AöR), Köln, Deutschland; 3 Stabsstelle Kaufmännische Direktion, Universitätsklinikum Köln (AöR), Köln, Deutschland Kerpener Straße 62, 50937

**Keywords:** Krankenhausplanung, Krankenhausfinanzierung, Krankenhausdichte, Krankenhausspezialisierung, Krankenhausdigitalisierung, Hospital planning, Hospital funding, Hospital density, Hospital specialization, Hospital digitalization

## Abstract

**Hintergrund:**

In den vergangen 30 Jahren hat sich in Deutschland die Anzahl der Krankenhäuser um 21 % und die Anzahl der Krankenhausbetten um 27 % reduziert. Im selben Zeitraum halbierte sich die durchschnittliche Verweildauer der Krankenhausfälle. Dennoch ist die Systemeffizienz zu gering.

**Fragestellung:**

Ursachen für die Systemineffizienz im Krankenhaussektor und Ansätze für eine Krankenhausreform.

**Material und Methode:**

Auswertung der amtlichen Statistiken, Zusammenfassung von Grundlagenarbeiten, Studien und Expertenempfehlungen sowie Übersichtsaufsätze.

**Ergebnisse:**

Das ordnungspolitische Nebeneinander von Krankenhausplanung und Krankenhausfinanzierung führt zu einer strukturellen Unterfinanzierung von Krankenhäusern und zu einer suboptimalen Versorgung mit Krankenhausleistungen. Das rein stationär bezogene Vergütungssystem konserviert stationäre Behandlungsformen. Deutschland weist im internationalen Vergleich eine sehr hohe Krankenhausdichte auf. Dies verschärft den Fachkräftemangel in den Gesundheitsfachberufen. Außerdem haben deutsche Krankenhäuser einen zu niedrigen Digitalisierungsgrad.

**Schlussfolgerung:**

Eine Reduzierung der Anzahl an Krankenhäusern mit gleichzeitig zunehmender Spezialisierung kann sowohl die Versorgungsqualität für die Patienten als auch die Effektivität des Einsatzes knapper Fachkräfte erhöhen. Es bedarf eines nach Versorgungsstufen differenzierten Finanzierungskonzepts. Eine Steigerung der Attraktivität von Gesundheitsfachberufen, insbesondere durch eine Verbesserung der Ausbildung, kann dem Fachkräftemangel begegnen. Das Krankenhauswesen muss mit der Digitalisierung aufschließen, um zeitgemäß ausgestattete Arbeitsplätze und modern organisierte Arbeitsprozesse anbieten zu können, auch, um die Wirtschaftlichkeit zu verbessern.

## Abstract (en)

**Background:**

Over the past 30 years the number of hospitals in Germany has fallen by 21% and the number of hospital beds by 27%. During the same period the average length of stay in hospitals has halved. Nevertheless, the system efficiency is too low.

**Objective:**

The causes of the system inefficiency in the German hospital sector and approaches for a hospital reform are presented.

**Material and methods:**

The official statistics were analyzed and a summary of fundamental works, studies and expert recommendations is given in a review article.

**Results:**

The regulatory coexistence of hospital planning and hospital financing leads to a structural underfunding of hospitals and to a suboptimal supply with hospital services. The purely inpatient-oriented remuneration system conserves inpatient forms of treatment. In an international comparison there is a high hospital density in Germany. This intensifies the shortage of specialist personnel in healthcare professions. In addition, the level of digitalization in German hospitals is too low.

**Conclusion:**

A reduction of the number of hospitals in conjunction with increased specialization can increase the quality of hospital treatment for the patients and also increase the effectiveness of the deployment of scarce specialist personnel. There is a need for a differentiated financing concept according to treatment levels. An increase in the attractiveness of specialist healthcare professions, particularly by improvement of the training can counteract the shortage of specialist personnel. The hospital system must catch up with digitalization in order to be able to provide up to date equipped workplaces and modern organized work processes and also to improve the economic efficiency.

Die deutsche Krankenhauslandschaft befindet sich seit Jahren im Umbruch. Die Anzahl der Krankenhäuser und Krankenhausbetten hat sich drastisch reduziert und eine Privatisierungswelle hat die Trägerstruktur deutlich verändert. Dennoch ist die Systemeffizienz zu gering. Das Krankenhausfinanzierungssystem führt zu einer suboptimalen Versorgung mit Krankenhausleistungen und bedingt eine strukturelle Unterfinanzierung von Krankenhäusern. Gleichsam verfügt Deutschland im internationalen Vergleich über eine sehr hohe Krankenhausdichte mit zu geringer Spezialisierung. Der Reformbedarf ist hoch.

## Der Krankenhausmarkt in Deutschland

**Figure 1:**
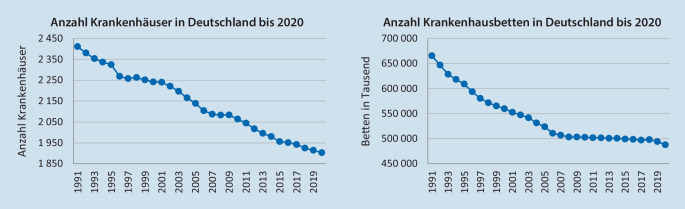

Der deutsche Krankenhausmarkt erreichte im Jahr 2020 ein Ausgabenvolumen von rund 114,1 Mrd. €, das sind 3,4 % des Bruttoinlandsprodukts und umfasste mit einem Anteil von 26 % das größte Volumen von allen Sektoren des deutschen Gesundheitswesens [15]. Im Jahr 2020 wurden in 1903 Krankenhäusern insgesamt 16,8 Mio. Fälle behandelt. Im Durchschnitt lag ein Fall 7,2 Tage im Krankenhaus. Hierfür hielt der deutsche Krankenhausmarkt rund 487.800 Betten bereit [17]. Bezogen auf die Einwohnerzahl mit 7,8 Betten je 1000 Einwohner fast doppelt so viele Betten wie der Durchschnitt der OECD-Staaten [8]. Dieser Vergleich ist interessant, verrät ein Blick in die Statistik, dass die Anzahl deutscher Krankenhäuser von 1991 bis 2020 bereits um 21 %, die Anzahl der Betten sogar um 27 % zurückgegangen ist (vgl. Abb. 1).

Gleichsam hat die Nachfrage nach Krankenhausleistungen im selben Zeitraum drastisch zugenommen. Blendet man den coronabedingten Fallzahlrückgang in 2020 aus und schaut auf die Entwicklung der Fallzahlen bis 2019, ist ein Anstieg von 33 % zu verzeichnen, wobei sich die durchschnittliche Verweildauer von 14 Tage auf 7,2 Tage fast halbiert hat [17]. Auch hier lohnt sich ein vertiefender Blick in die Statistik. Obwohl sich die Anzahl der Betten seit 1991 um mehr als ein Viertel reduziert hat, ist die Auslastung trotz drastisch gestiegener Fallzahlen rückläufig. Während die Betten 1991 mit 84,1 % nahezu voll ausgelastet waren, lag die Auslastung in 2019 nur noch bei 77,2 %, was nahelegt, dass in Deutschland noch immer zu viele Krankenhausbetten beziehungsweise Krankenhäuser betrieben werden.

### Krankenhausplanung

Die Planung von Krankenhauskapazitäten liegt in der Verantwortung der Bundesländer. Nach dem Krankenhausfinanzierungsgesetz (§ 1 in Verbindung mit § 6 KHG) haben sie den Auftrag, hinreichende Kapazitäten für eine qualitativ hochwertige, patienten- und bedarfsgerechte sowie wirtschaftliche Versorgung der Bevölkerung mit Krankenhausleistungen sicherzustellen. Das allgemein übliche Vorgehen, die Krankenhausplanung am Parameter Bett sowie einer groben Rahmenplanung von Fachgebieten auszurichten, ist wegen des fehlenden Leistungsbezugs umstritten. Kritisiert werden insbesondere mangelnde Kontroll- und Steuerungsmöglichkeiten über das tatsächliche Leistungsgeschehen in den vorgehaltenen Betten.

Kritisiert werden insbesondere mangelnde Kontroll- und Steuerungsmöglichkeiten

Weitere Aspekte, die für eine Abkehr von der Planungsgröße Bett sprechen, sind Fehlanreize in der Verweildauersteuerung, die unzureichende Abbildung des medizinischen Fortschritts, der alleinige Bezug zur stationären Versorgung sowie ein zu geringer Qualitätsbezug zur Leistungserbringung [9]. Nordrhein-Westfalen hat in 2021 als erstes Bundesland beschlossen, die Bettenplanung aufzugeben. Künftig sollen in der Krankenhausplanung des Landes Nordrhein-Westfalen Leistungsbereiche mit Leistungsgruppen ausgewiesen werden [10].

### Versorgungsstufen

Die Kategorisierung der Krankenhäuser hinsichtlich ihrer Beteiligung an Art und Umfang der Versorgung mit Krankenhausleistungen variiert von Bundesland zu Bundesland. Einige Bundesländer differenzieren im Allgemeinen nach drei oder vier Versorgungsstufen (beispielsweise Grund‑, Regel und Maximalversorgung). Die meisten Bundesländer kategorisieren jedoch ihre Krankenhäuser nach anderen Kriterien, wie zum Beispiel nach Versorgungsgebiet, Gesamtzahl der Planbetten, die Art der Abteilungen mit ihrer Planbettenzahl und ihren Behandlungsplätzen.

Der Koalitionsvertrag aus dem Jahr 2021 sieht vor, die Planung von Krankenhauskapazitäten in ganz Deutschland neu aufzustellen und in diesem Zuge unter anderem auch eine Angleichung der Krankenhauskategorisierung zwischen den Bundesländern herbeizuführen. So hat eine von der Bundesregierung einberufene Expertenkommission den Auftrag, „Leitplanken für eine auf Leistungsgruppen und Versorgungsstufen basierende und sich an Kriterien wie der Erreichbarkeit und der demographischen Entwicklung orientierende Krankenhausplanung“ [5] zu erarbeiten.

### Krankenhausträger

**Figure 2:**
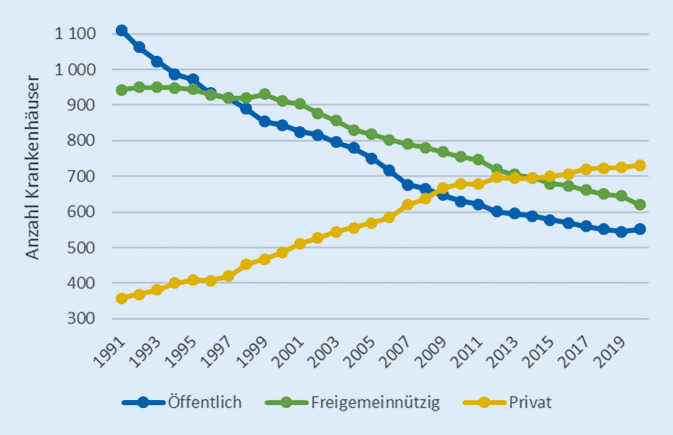

Die deutsche Krankenhauslandschaft ist von einer historisch gewachsenen und durch das Krankenhausfinanzierungsgesetz (§ 1 KHG) gesetzlich geschützten Vielfalt an Krankenhausträgern geprägt. In den vergangenen drei Jahrzehnten hat in der Trägerstruktur ein tief greifender Wandel stattgefunden. Im Zuge der sogenannten Krankenhausprivatisierungswelle haben sich beachtliche Marktanteile von der öffentlichen bzw. freigemeinnützigen zur privaten Trägerschaft verlagert (vgl. Abb. 2).

Während die Anzahl der freigemeinnützigen Häuser um ein Drittel abnahm und sich die Anzahl der öffentlichen Krankenhäuser sogar mehr als halbierte, verdoppelte sich die Anzahl der Krankenhäuser in privater Trägerschaft. Übersetzt in Marktanteile heißt dies, dass die privaten Träger ihren Marktanteil von 14,8 % in 1991 auf 38,5 % in 2020 ausbauen konnten, währenddessen der Marktanteil der öffentlichen Träger von 46 % auf 29 % geschrumpft ist. Ein Blick auf die Bettenverteilung relativiert das Marktverhältnis. 2020 befand sich jedes zweite Bett in öffentlicher und nur jedes fünfte in privater Hand [17].

## Strukturelle Probleme in der Krankenhauslandschaft

### Hohe Krankenhausdichte

2020 gab es in Deutschland noch 1903 Krankenhäuser mit 487.783 Betten und einer Auslastung von nur 67,3 % [16]. Die Auslastung dürfte zwar wegen Covid-19 in 2021 und 2022 angestiegen sein, aber es gibt nach wie vor zu viele und meist zu kleine Krankenhäuser in Deutschland. Nur 95 Krankenhäuser haben mehr als 800 Betten.

Die Dichte von Krankenhausbetten je 1000 Einwohner reicht 2020 von 488 in Baden-Württemberg bis 741 in Bremen [13]. Allein daraus wird deutlich, dass es in Deutschland zumindest in einigen Bundesländern zu viele und zu kleine Krankenhäuser gibt, die den modernen Anforderungen im Hinblick auf genügend Fallzahlen, moderne Technikausstattung und Fachpersonal nicht gerecht werden und gerecht werden können.

Es gibt nach wie vor zu viele und meist zu kleine Krankenhäuser

Die Bertelsmann-Stiftung hat dies am Beispiel NRW schon 2019 eindeutig belegt und macht entsprechende Strukturveränderungsvorschläge für 2030 mit einem zweistufigen Versorgungsstufenkonzept [3].

Bei dem vorgeschlagenen qualitätsbasierten Vorgehen werden in einem ersten Schritt die Krankenhäuser der Regelversorgung ausgewählt, die im Status quo bestimmte Anforderungen an die Strukturqualität für die Notfallbehandlung von Patienten mit Herzinfarkt oder Schlaganfall erfüllen. Bei der Auswahl der Standorte der Maximalversorger werden die Bevölkerungszahl in der Versorgungsregion sowie die Größe und raumplanerische Bedeutung der Städte in der Region berücksichtigt. Anschließend werden die Erreichbarkeiten der Regel- und Maximalversorger berechnet. Schließlich werden die für 2030 geschätzten Behandlungsfälle auf die Standorte verteilt und die resultierenden Standort- und Fachabteilungsgrößen berechnet. Abschließend wird die Einhaltung weiterer Qualitätsanforderungen geprüft: Zum einen, ob die derzeit in den Krankenhäusern der Versorgungsregion tätigen Fachärzte ausreichen, um auch im qualitätsbasierten Versorgungsmodell eine ständige fachärztliche Versorgung sicherzustellen, – zum anderen, ob die für die Notfallversorgung von Herzinfarktpatienten ausgewählten Krankenhäuser die entsprechende Mindestmenge im Status quo erfüllen bzw. künftig erfüllen können.

Nur durch ein solches Vorgehen kann eine qualitativ deutlich bessere Krankenversorgung mit gleichzeitig mindestens 20 % weniger Krankenhäusern sichergestellt werden.

### Mangelnde Spezialisierung

**Figure 3:**
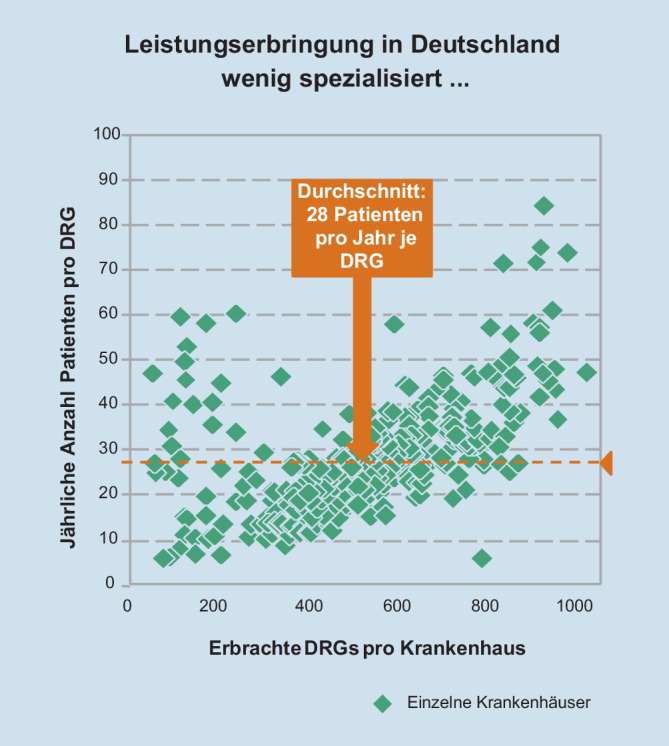

Einher mit der zu hohen Krankenhausdichte geht die mangelnde Spezialisierung: Es sollte unmittelbar einsichtig sein, dass – wie in Abb. 3 dargestellt – im Durchschnitt nur etwa 30 Fälle einer Erkrankung pro Jahr in einer Klinik bei einer Behandlung weit weniger erfolgreich sind, gegenüber einer spezialisierten Klinik oder Fachabteilung mit im Durchschnitt mehreren Hundert Fällen. Ein besonders eindrucksvolles Beispiel ist die Wiederholungs- und Inkontinenzrate bei Prostata-Operationen. Diese ist zum Beispiel in der Martini-Spezialklinik in Hamburg mit weit über 2000 Operationen pro Jahr um mehr als 50 % geringer als in Kliniken mit 30–50 Prostata-Operationen pro Jahr [7].

Das Beispiel zeigt, dass nur durch eine ausreichende Spezialisierung nach Krankheiten eine optimale und zugleich effizientere Krankenversorgung gewährleistet wird. Stellt man dabei eine regionale Betrachtungsweise in den Vordergrund, so wird deutlich, dass eine krankenhausträgerübergreifende Spezialisierung anzustreben wäre, aber aufgrund der unterschiedlichen Eigentümerinteressen wohl nur sehr schwer umsetzbar ist.

### Zunehmender Fachkräftemangel

Durch die Verteilung von zu wenigen Fachkräften auf zu viele Krankenhäuser wird das allgemein in Deutschland vorhandene Problem des Fachkräftemangels vor allem in der Pflege nochmals deutlich verschärft. Covid-19 hat hier zusätzlich als „Brandbeschleuniger“ gewirkt und viele Pflegekräfte sind primär wegen der extremen Arbeitszeiten in andere Berufe abgewandert. Neben den bereits eingeleiteten Maßnahmen wie bessere Entlohnung, mehr Freizeit und flexiblere Arbeitszeiten dürfte mittelfristig nur eine Bündelung der Kräfte zu einer ausreichenden Versorgung führen. So begünstigen weniger, dafür aber größere Krankenhäuser die Entwicklung neuer Arbeitszeitmodelle für die Branche Krankenhauswesen. Die Bildung von gemeinsamen Ausbildungszentren schafft Synergien für das Lehrpersonal und fördert neben einem breiteren Angebot an Ausbildungsplätzen für Gesundheitsfachberufe auch die interprofessionelle Ausbildung. Auch muss die Gewinnung neuer Pflegekräfte aus anderen Berufen und dem Ausland weiter ausgebaut werden. Aktuellen Studien zufolge werden 2030 rund 461.300 und 2035 rund 493.600 Pflegekräfte benötigt, was einem Anstieg bis 2035 gegenüber heute von rund 31 % entspricht [14].

Der Fachkräftemangel betrifft aber nicht nur die Pflege. Auch für die notwendige Digitalisierung in den Krankenhäusern werden in jeder Klinik Fachkräfte benötigt, sodass auch hier ein Mangel bis 2030 von mindestens 10.000 Fachkräften vorhanden ist.

Der Fachkräftemangel wird massiv zunehmen

Und auch bei den Ärzten zeichnet sich ein massives Nachfolgeproblem ab: Heute sind nicht zuletzt auch wegen des vollkommen überzogenen Numerus clausus für Medizin beinahe 70 % der Medizinstudierenden weiblich. Das ist aus Gleichheitsgesichtspunkten sicherlich zu begrüßen, bedeutet aber in etwa zehn Jahren die Notwendigkeit, beinahe jede Arztstelle doppelt zu besetzen, um so Schwangerschafts- und Kindererziehungszeiten zu kompensieren. Folglich müsste eigentlich die Anzahl der Medizinstudienplätze zeitnah verdoppelt werden, was momentan in keinem Bundesland geplant ist.

Es zeichnet sich also bereits heute ab, dass der Fachkräftemangel in deutschen Krankenhäusern bei medizinischem und pflegendem Personal massiv zunehmen wird und die Politik hier dringend strukturelle Gegenmaßnahmen einleiten muss. Diese müssen bei Ausbildungs- und Studienplätzen ansetzen und erfordern eine langfristige Personalplanung in den Krankenhäusern auf Grundlage der zukünftigen quantitativen und qualitativen Bedarfe. Nur wenige Krankenhäuser haben bisher eine solch umfassende und langfristige Modellierung ihrer Personalbedarfe vorgenommen. Das Beispiel der Ergebnisse einer solchen Modellrechnung an der Universitätsklinikum Köln hat gezeigt, dass die durch altersbedingte Abgänge und neue Bedarfe entstehenden Lücken bereits heute nur noch durch ein umfangreiches Maßnahmenprogramm geschlossen werden können.

### Unzureichende Finanzierung

Instandhaltungsbudgets auf dem Niveau der 1970er-Jahre einerseits sowie insbesondere für Maximalversorger nicht auskömmlicher Fallpauschalen andererseits treiben die Krankenhäuer schon seit Jahren in eine – sich kumulierende – strukturelle Unterfinanzierung. Diese Entwicklung wird nun nochmals deutlich verschärft durch die steigenden Personalzahlen in der Pflege, die signifikanten Lohnsteigerungen und die Inflation bei den Energie- und Gebäudekosten. So müssen Krankenhäuser für 2023 etwa mit den dreifachen Energiekosten gegenüber heute rechnen [12].

Es werden sich also im Jahr 2023 Verluste in den heute rund 1900 deutschen Krankenhäuser anhäufen, die sicherlich bundesweit in Summe bei mindestens € 8 Mrd. liegen dürften. Diese Belastung wird viele Träger an den Rand der Insolvenz führen. Der Staat wird sicher nicht alle davon betroffenen Krankenhäuser retten können, aber vielleicht liegt hierin auch die Chance, die jahrzehntelang verschleppte Krankenhausreform nun unter dem Druck des Faktischen auch tatsächlich umzusetzen.

Steigende Kosten werden viele Träger an den Rand der Insolvenz führen

Weniger spezialisierte und regional vernetzte Krankenhäuser können alle zuvor genannten Probleme besser lösen: Durch mehr Effizienz und Größenvorteile sowie gleichzeitig gestiegene Versorgungsqualität können Personalmangel und Kostensteigerungen wesentlich besser adressiert werden, ohne dass die Kassen mit Ausnahme der höheren Fallpauschalen viel mehr als heute bezahlen müssen. Ferner sollte ein künftiges Vergütungssystem auch die in anderen Ländern schon länger umgesetzte Praxis der ambulanten Durchführung von elektiven Operationen vorsehen, um so auch die Nachfrage nach Krankenhausaufenthalten zu reduzieren.

Diese notwendigen strukturellen Optimierungsmaßnahmen bei der Finanzierung dürfen aber nicht darüber hinwegtäuschen, dass die überfällige Modernisierung von Gebäuden, medizinischen Geräten und IT-Infrastrukturen in den Krankenhäusern zu deutlich höheren Instandhaltungsrückstellungen und -kosten führen werden, um nicht in zehn oder 20 Jahren erneut vor einem Investitionsstau zu stehen.

Insgesamt bleibt also festzuhalten, dass im Rahmen einer Krankenhausreform zwingend ein nach Versorgungsstufen und Qualitätskriterien differenziertes, neues Finanzierungskonzept eingeführt werden muss, das für alle Träger auf Bundes- Landes- oder kommunaler Ebene gleichermaßen Anwendung findet und die notwendigen Investitionen in die Modernisierung der Krankenhauslandschaft berücksichtigt.

### Noch weitgehend fehlende Digitalisierung

Bisher hat die Digitalisierung in Krankenhäusern wenig Einzug gehalten. In den meisten Kliniken reduziert sie sich lediglich auf eine WLAN-Abdeckung. Die Pandemie hat den Digitalisierungsbedarf der Ärzte offengelegt. Sie wollen in Kliniken und Praxen mehrheitlich Digitaltechnik wie Videosprechstunden, künstliche Intelligenz (KI) zur Diagnose oder virtuelle Realität beim Training für Operationen einsetzen, hat eine Umfrage des deutschen Digitalverbands Bitkom und des Ärzteverbands Hartmannbund ergeben [4].

Die realen Arbeitsbedingungen im Klinikalltag sind dagegen weitgehend analog: Alles wird auf Papier dokumentiert. Eine elektronische Patientenakte ist meist nicht vorhanden und die tägliche Visite erfolgt mit Papierakten oder fahrbarem Computergestell.

Sechs von zehn Ärzten kommunizieren weiter per Fax, hat die Studie ermittelt. Weniger als ein Fünftel der Ärzteschaft bietet Videosprechstunden an. Nicht einmal jede zehnte Klinik arbeitet mit KI [4].

Fast alle 535 bundesweit befragten Ärzte sehen die Bürokratie als Haupthindernis, acht von zehn eine mangelnde Marktreife digitaler Produkte, sieben von zehn auch einen ihrer Meinung nach übertriebenen Aufwand für Datenschutz.

Nur 6 % der Ärzte nutzen elektronische Patientenakten

Auch bei zwei Hoffnungsträgern digitaler Medizin geht es kaum oder nur schleppend voran. Gerade einmal 5 % der Ärzte haben in Deutschland schon einmal ein elektronisches Rezept ausgestellt, 6 % nutzen elektronische Patientenakten. Jeweils ein Fünftel sagt, beides auch künftig nicht nutzen zu wollen, wobei die elektronische Akte zudem von 13 % aller Patienten abgelehnt wird [4].

Der Bitkom sieht in Digitalmedizin ein wirksames Mittel zur Kostensenkung im Gesundheitswesen und für passgenaue individuelle Therapien. Dazu müssten vorhandene Gesundheitsdaten besser nutzbar gemacht werden, was derzeit vielfach am Datenschutz scheitert [4].

Um eine vollständige elektronische Patientenakte mit allen bildgebenden Verfahren und Labordaten in einem Krankenhaus in allen Abteilungen und Stationen einzuführen, bedarf es massiver Investitionen in Software und Infrastruktur. Auch wenn es dafür Fördertöpfe gibt, stellt auch hier vor allem der IT-Fachkräftemangel in den Krankenhäusern das größte Hindernis da. Daher wird es wohl noch Jahre dauern, bis eine breitflächige Digitalisierung in den Krankenhäusern in Deutschland erreicht sein wird.

## Umfassende Krankenhausreform notwendig

**Figure 4:**
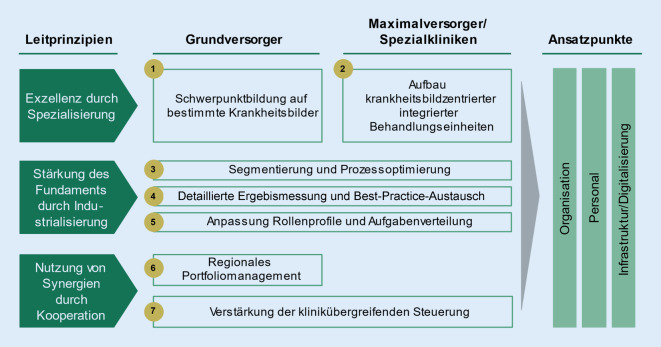

Wie die zuvor dargestellten Ausführungen belegen, bedarf es einer umfassenden Krankenhausreform in Deutschland, um alle bekannten Probleme gesamthaft zu adressieren. In Anlehnung an die in Abb. 4 dargestellte Value-Based-Healthcare-Methodik sollte die Reform sieben Handlungsfelder adressieren [2]:

Drei Leitprinzipien prägen dabei die notwendigen strukturellen Veränderungen.

Durch eine regionale, über Versorgungsstufen hinweg organisierte Spezialisierung werden die Fallzahlen und damit die Versorgungsqualität spürbar erhöht und gleichzeitig die Effizienz gesteigert, da vor allem in größeren, spezialisierten Einheiten das benötigte Fachpersonal flexibler eingesetzt und die teuren Geräte besser genutzt werden können. In diese Überlegungen sind unbedingt auch ambulante OP-Praxen mit einzubeziehen.

Damit einhergehen sollte eine umfassende Prozessoptimierung in den Krankenhäusern, die vor allem auch eine interdisziplinäre Zusammenarbeit nach Krankheitsbildern statt nach Fachabteilungen zum Ziel hat.

Organisation, Personalportfolio und die Infrastruktur sowie IT müssen diese Prozessveränderungen in vollem Umfang unterstützen. Es bedarf einer Neuorganisation des gesamten Krankenhauses und der Digitalisierung aller Prozesse, um die Anzahl der Schnittstellen zu minimieren.

Es bedarf einer Neuorganisation des gesamten Krankenhauses

Als letzter Schritt wäre eine trägerunabhängige regionale Kooperation zwischen allen Kliniken und niedergelassenen Ärzten anzustreben, um das System als Ganzes zu optimieren.

### Weniger Krankenhäuser mit umfassender Spezialisierung

Wie das Beispiel der Bertelsmann-Studie für die Region um Köln zeigt (vgl. Tab. 1), könnte die heutige Anzahl von 38 Krankenhäusern auf 14 spezialisierte Grundversorger und vier Maximalversorger reduziert werden, ohne dass darunter die Versorgungsqualität leiden würde [3]:

**Table 1:** 

Anzahl erforderlicher Krankenhausstandorte in der Versorgungsregion 5 gemäß Zielmodell und Erreichbarkeit, 2030	
**Max. 30** **min (Regelversorgung)**	
*Anzahl Standorte*	**14**
*Mittlere Fahrzeit in Minuten*	17
*Anteil Bevölkerung mit Fahrzeiten*	
Länger als 30 min	3,0 %
15 bis 30 min	49,0 %
Weniger als 15 min	48,0 %
**Max. 60** **min (Maximalversorgung)**	
*Anzahl Standorte*	**4**
*Mittlere Fahrzeit in Minuten*	24
*Anteil Bevölkerung mit Fahrzeiten*	
> 60 min	0,0 %
> 45 bis ≤ 60 min	0,5 %
> 30 bis ≤ 45 min	14,8 %
> 15 bis ≤ 30 min	66,3 %
≤ 15 min	18,3 %

Datenquelle: Bertelsmann-Stiftung: Zukunftsfähige Krankenhausversorgung, Juli 2019 [3]

Auch wenn sich sicher Gegenargumente gegen einzelne Annahmen der Studie finden lassen, so macht sie doch für die wichtigsten Krankheitsbilder heute und in Zukunft deutlich, dass es in Deutschland zu viele, zu kleine und zu wenig ausgelastete und spezialisierte Krankenhäuser gibt. Hierin liegt der wohl bedeutsamste Ansatzpunkt einer Krankenhausreform.

Erst nach dieser Strukturbereinigung und der Stärkung ambulanter Spezialpraxen für elektive Operationen kann eine organisatorische, personelle und infrastrukturelle Neuausrichtung der dann noch existierenden Standorte beginnen und die ebenfalls notwendigen Prozess- und IT-Optimierungen im Sinne einer Patientenzentrierung umgesetzt werden. Andernfalls würde man lediglich alte Strukturen stärker digitalisieren, ohne die gewünschten Qualitäts- und Kostenwirkungen zu erzielen.

### Differenzierte Finanzierungsmodelle nach Versorgungsstufen

In einer Pressemitteilung vom Juni 2022 fasst das RWI-Leibniz-Institut für Wirtschaftsforschung die Ergebnisse des Krankenhaus-Rating-Reports 2022 zusammen. Dieser empfiehlt, Vergütungsinstrumente auf Systemebene anzusetzen. Zwar hat sich die Finanzierung von Krankenhausleistungen seit Einführung des DRG-Systems verbessert, doch trägt dies nicht zwangsläufig zu einer Effizienzverbesserung im Gesundheitswesen bei. So motiviert die auf Durchschnittskosten basierende DRG-Vergütung zu ökonomischen Mengenausweitungen. Zudem verhindert der ausschließlich stationäre Bezug die Entwicklung von sektorübergreifenden Versorgungskonzepten sowie die Ambulantisierung der Medizin [11].

Der Verband der Deutschen Universitätsklinika tritt für ein Finanzierungskonzept nach Versorgungsstufen ein, weil die durchschnittskostenbasierenden DRG-Vergütungen zu einer eklatanten strukturellen Unterfinanzierung von Maximalversorgern und Universitätsklinika führen. In Krankenhäusern der Maximalversorgung wird eine hohe Anzahl von komplexen und hochaufwendigen Erkrankungen mit nur geringen Fallzahlen behandelt. Dies erfordert die Vorhaltung von sehr kostenintensiven Strukturen, wie sie sich beispielsweise aus der verpflichtenden Umsetzung von G‑BA-Vorgaben ergeben [18]. Diese Kostenkomponenten sind nicht hinreichend in den DRG berücksichtigt. Ein Blick in den InEK-Abschlussbericht 2020 zeigt, dass die hohen Vorhaltekosten von Maximalversorgern schon rein mathematisch nicht adäquat in dem Vergütungssystem abgebildet sein können. Von den Häusern, die sich an den Kalkulationen beteiligen, verfügen rund 85 % über weniger als 600 Betten [6].

Das DRG-System verhindert Effizienzverbesserungen im Gesundheitswesen

Die im Koalitionsvertrag aus dem Jahr 2021 angestrebte Weiterentwicklung der Krankenhausplanung und -finanzierung zielt auf die Verbindung der Krankenhausplanung mit der Krankenhausfinanzierung ab. Zum einen soll eine Krankenhausplanung auf Basis von Leistungsbereichen und Leistungsgruppen dafür Sorge tragen, dass in den zur Verfügung stehenden Krankenhauskapazitäten die Leistungen erbracht werden, welche die Bevölkerung benötigt und nicht die, die für ein Krankhaushaus betriebswirtschaftlich lukrativ sind. Zum anderen führt die angestrebte Erweiterung der Krankenhausfinanzierung nach Versorgungsstufen differenzierte Vorhaltepauschalen ein. Gleichzeitig wird die Ambulantisierung von Krankenhausleistungen, die vorrangig nur in diesen Strukturen erbracht werden können, aber keiner stationären Aufnahme bedürfen, diskutiert. Durch eine sektorengleiche Vergütung soll die Kostenstruktur im Krankenhausbereich differenzierter abgebildet und damit vergütet werden.

Allerdings greift eine Krankenhausreform, die sich nur auf die Finanzierung von Personal- und Sachkosten konzentriert, zu kurz. Solange nicht auch die Investitionsfinanzierung hinreichend gesichert ist, werden die Krankenhäuser nach wie vor in die Unwirtschaftlichkeit gedrängt, zum Beispiel, weil mit einem überalterten Gerätepark hohe Wartungskosten oder Aufwendungen für Abschreibungen anfallen, für deren Finanzierung keine Anteile in die DRG einkalkuliert sind.

### Bessere Ausbildungsanreize für Gesundheitsfachberufe

Eine Konsolidierung der Krankenhauslandschaft wird das Problem der fehlenden Fachkräfte mildern, aber nicht lösen. Ziel muss es daher sein, junge Menschen für die Ausübung eines Gesundheitsfachberufs zu begeistern.

**Figure 5:**
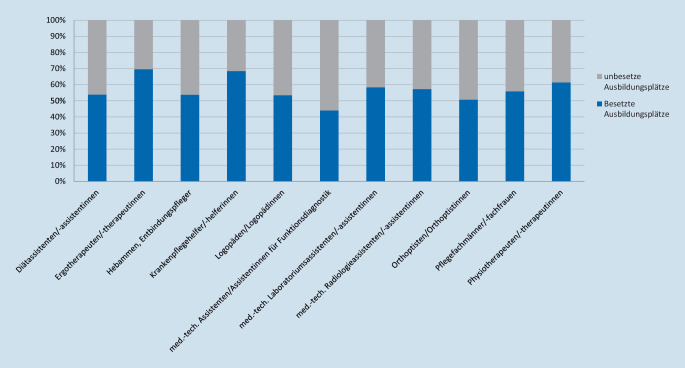

Hier hat Deutschland enormen Aufholbedarf. Allein im Jahr 2020 blieben in zahlreichen Gesundheitsfachberufen fast die Hälfte der verfügbaren Ausbildungsplätze wegen Bewerber- und Fachkräftemangel unbesetzt (vgl. Abb. 5; [17]).

Vor dem Hintergrund des schrumpfenden Erwerbstätigenpotenzials wird das Gesundheitswesen bei der Gewinnung von Auszubildenden mit anderen Branchen konkurrieren. Um sich für einen Gesundheitsfachberuf zu entscheiden, brauchen junge Menschen eine attraktive Perspektive. Insbesondere ist das öffentliche Bild des Pflegeberufs stark beschädigt. Es wird mit geringer Wertschätzung und hoher Arbeitsbelastung verbunden, was im Laufe des Erwerbslebens zu Frustrationen führt und häufig in Arbeitszeitreduzierungen oder gar vorzeitigem Berufsausstieg endet. Dieses Bild gilt es abzulösen durch die Etablierung von Karrierewegen mit klaren Rollenbildern, sodass sich jungen Menschen neue Visionen auftun.

Doch zuvorderst brauchen junge Menschen ein ansprechendes Ausbildungsangebot, denn die Attraktivität eines Berufes beginnt bei der Ausbildung. Dies erfordert die Etablierung von modernen, interprofessionellen Lernkonzepten, die erstens der medizin-technischen Entwicklung Rechnung tragen und zweitens auf die Bedürfnisse der Lernenden und Lehrenden abgestimmt sind.

Junge Menschen brauchen ein ansprechendes Ausbildungsangebot

Mit einer guten technischen Ausstattung der Ausbildungseinrichtungen wie beispielsweise mit SkillLabs, Lerninseln, Videokonferenzen und mit der Verfügbarkeit einer zeitgemäßen Digitalisierung kann die Qualität der Ausbildung nachhaltig verbessert werden. Ergänzende E‑Learning-Angebote antworten auf die digitale Welt junger Menschen. Sie fördern das eigenständige, unabhängige Lernen und sichern die Sofortverfügbarkeit von Unterrichtsmaterialen. Digitale Bibliotheken mit einem ausreichenden Angebot an Endgeräten runden den Zugang zum Wissen ab.

Attraktive Ausbildungsvoraussetzungen können mit einem hochdifferenzierten Angebot an Aus- und insbesondere Weiterbildungsmöglichkeiten in speziellen Fachgebieten geschaffen werden, mit dem Anspruch, eine qualitativ hochwertige Aus- und Weiterbildung von der Regelversorgung bis hin zur Hochleistungsmedizin sicherzustellen. Ein großes Spektrum an Berufsoptionen eröffnet persönliche Karrierechancen, was vorzeitigen Berufsausstiegen entgegenwirkt. So gilt es zum Beispiel, den Pflegekräften in Krankenhäusern und anderen Einrichtungen zukünftig ergänzend zur klassischen Führungslaufbahn auch neue Rollen in einer fachlichen und pädagogischen Berufsausrichtung zu ermöglichen.

### Verkürzte Anerkennungszeiten für ausländische Fachkräfte

Das am 1. März 2020 in Kraft getretene Fachkräfteeinwanderungsgesetz regelt, dass ausländische Fachkräfte mit einem akademischen Abschluss oder einer Berufsausbildung mit einer Anerkennung ihrer ausländischen Qualifikation in Deutschland arbeiten dürfen. Ebenso entfällt die sogenannte Vorrangprüfung, wonach bei einer Bewerbung von Fachkräften aus Drittstaaten zunächst geprüft wird, ob ein Arbeitsplatz mit Arbeitskräften aus Deutschland oder der EU besetzt werden könnte. Diese Neuregelungen sollen es Arbeitgebern erleichtern, internationale Fachkräfte zu akquirieren, um einen Defizitausgleich des heimischen Fachkräftemangels herbeizuführen. Dabei geht es darum, internationale Fachkräfte so schnell wie möglich einsetzen zu können, das heißt konkret, den Einwanderungsprozess und die für eine Visumserteilung erforderlichen Berufsanerkennungsverfahren zu verkürzen.

Das beschleunigte Fachkräfteverfahren nach § 81a AufenthG soll das Verwaltungsverfahren von der Zusage der Fachkraft bis zum ersten Arbeitstag durch strukturierte Abläufe sowie vorgegebene Fristen verkürzen.

Im Zuge der Umsetzung dieses Gesetzes wurden auf Landesebene entsprechende Unterstützungsstellen wie zum Beispiel in Nordrhein-Westfalen die Zentralstelle Fachkräfteeinwanderung Nordrhein-Westfalen (ZFE NRW) implementiert. Diese Behörde bietet ein Online-Dokumentenmanagement an. Durch diese digitalisierte und verkürzte Antragstellung garantiert die ZFE NRW die Ausstellung des Aufenthaltstitels mit Arbeitserlaubnis binnen acht Wochen. Aber auch vor den Erleichterungen des Fachkräfteeinwanderungsgesetztes gab es strukturierte Programme zur Gewinnung von Fachkräften. So entstand im Jahr 2013 durch die Zusammenarbeit der Deutschen Gesellschaft für Internationale Zusammenarbeit (GIZ) und der Zentralen Auslands- und Fachvermittlung (ZAV) der Bundesagentur für Arbeit das sogenannte Triple-Win-Programm für die Gewinnung von Pflegekräften aus Drittstaaten.

Darüber hinaus kann die Etablierung krankenhauseigener Strukturen den Prozess nochmals beschleunigen. Hier liegen die Beschleunigungschancen insbesondere im Anerkennungsverfahren. Bewerber aus Drittstaaten haben die Möglichkeit, die Anerkennung ihrer Berufsqualifikation entweder über eine Kenntnisprüfung oder durch einen Anpassungslehrgang zu erwirken [1]. So kann das rekrutierende Krankenhaus beispielsweise beim Erwerb der erforderlichen Sprachnachweise oder bei der Vorbereitung auf Prüfungen mit Kursen in hauseigenen Pflegeschulen unterstützen und mit geeigneten Strukturen den zeitlichen Ablauf im Rahmen des Anpassungslehrgangs, in dem in fünf Bereichen Theorie- und Praxisstunden nachgeholt werden müssen, optimieren.

### Digitalisierung aller dafür geeigneten Arbeitsabläufe

**Figure 6:**
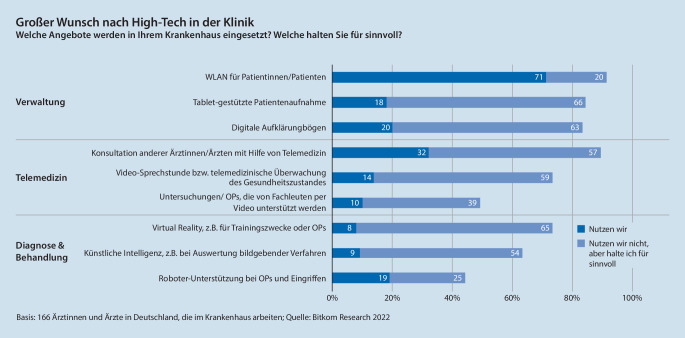

Wie zuvor dargestellt, müssen zusammen mit dem Digitalisierungsbestreben der Krankenhäuser (vgl. Abb. 6) die Prozesse in den Kliniken rund um den Patienten neu gestaltet werden. Erst dann kommt zum Beispiel eine elektronische Patientenakte voll zu einer für das Klinikpersonal entlastenden Wirkung.

Der Einsatz von Telemedizin ist in Krankenhäusern nur punktuell sinnvoll, z. B. um alle notwendigen Spezialisten schnell zusammen zu bringen wie bei einem Tumorboard. Die tägliche Visite ist dafür eher weniger geeignet. Für niedergelassene Ärzte, insbesondere auf dem Land, bietet die Telemedizin dagegen umfangreiche Effizienzpotenziale und verbessert gleichzeitig die qualitative Patientenversorgung. Gerade für ältere, gehbehinderte Menschen und in ländlichen Gebieten kann die Telemedizin in Verbindung mit App-basierten Gesundheitsdaten von Sensoren zu einer spürbaren Qualitätsverbesserung für Patienten führen.

VR, KI und Big Data kommen vor allem bei der klinischen Forschung und bei der Therapiebildung zum Einsatz. Der Covid-19-Impfstoff von BionTech konnte nur so schnell entwickelt und frei gegeben werden, weil statt jahrzehntelanger klinischer Studien mithilfe von Big Data und KI Millionen von Datensätzen auf die Wirkungszusammenhänge und potenziellen Nebenwirkungen hin analysiert wurden. Der Genauigkeitsgrad dieser Datenanalysen ist dem von manuell erfassten klinischen Studiendaten deutlich überlegen.

Natürlich muss aber in jeder Klinik die notwendige und sichere WLAN-Infrastruktur bereitgestellt werden. Künftig erwarten Patienten nicht nur einen Internetzugang in ihrem Zimmer, sondern auch die reibungslose Übertragung ihrer Gesundheitsdaten zum Beispiel von Wearables an den sie behandelnden Arzt.

## Fazit und Ausblick

Die vorangegangenen Ausführungen machen mehr als deutlich, dass es in Deutschland nach über 20 Jahren eine allumfassende Krankenhausreform bedarf, um die strukturelle Unterfinanzierung durch die Fallpauschalen gerade auch der Maximalversorger zu beseitigen, die Versorgungsqualität für die Patienten spürbar zu verbessern und dem Fachkräftemangel zu begegnen. Dabei muss es diesmal gelingen, zu bundeseinheitlichen und trägerübergreifenden Regelungen zu kommen. Auch die Verlagerung auf ambulante Operationspraxen spielt eine wesentliche Rolle zur Kostensenkung und Qualitätsverbesserung. Die Krankenversicherungen müssen in Zukunft diese Behandlungsmöglichkeiten incentivieren und erstatten.

Die für diese Reform durch die Politik zu überwindenden Hürden sind enorm hoch: Allein die Bundesländer zu einer einheitlichen Reform zu vereinen, dürfte schwierig sein. Noch schwieriger ist die Überzeugung von Landräten, Kommunen und Personalvertretungen zur notwendigen Schließung von Standorten. Und schließlich bedarf es auch noch der Zustimmung der verschiedenen Krankenhaus- und Kostenträger zu einer engeren Zusammenarbeit bei der Spezialisierung und der Kostenerstattung ambulanter Operationen.

Vor uns liegt also ein Kraftakt, der es aber vor allem im Interesse der Patienten und Bürger Wert ist, ihn mit aller Macht anzugehen. Die ebenso überfällige Digitalisierung aller Kernprozesse im Gesundheitswesen hat gezeigt, wie stark die Widerstandskräfte von allen Beteiligten sind. Auch hier wird man nur Fortschritte erzielen, wenn Deutschland die vorhandenen Technologieinnovationen zulässt und sich endlich wie andere EU-Staaten den europäischen Datenschutzstandards anpasst, anstatt diese noch deutlich zu verschärfen.

Wie viel einfacher könnte doch für alle Bürger und Beschäftigte im Gesundheitswesen das Leben im Jahr 2030 sein, wenn jeder seine persönlichen Gesundheitsdaten online und überall verfügbar hätte, jeder wüsste, wo er in seiner Nähe mit seiner Erkrankung den besten Spezialisten findet, dort online einen Termin über ein praxis- und krankenhausübergreifendes Portal bekommt, seine persönlichen Daten ohne aufwendige Anmeldeprozesse digital hinterlegen kann und dann auch noch die Medikation und Abrechnung automatisch erfolgt. All dies wäre bereits heute technisch möglich, aber geltende Regelungen bremsen diesen Digitalisierungsprozess. Gleichsam kommt der Staat an seine Grenzen, ein strukturell veraltetes System am Leben zu halten und zu finanzieren.

Die Hoffnung aller Beteiligten beruht daher auf einem baldigen Gelingen der vom Bundesgesundheitsministerium angekündigten großen Krankenhausreform.

## Fazit für die Praxis

- Weniger Krankenhäuser mit umfassender Spezialisierung erhöhen sowohl die Versorgungsqualität für die Patienten als auch die Effektivität des Einsatzes von knappen Fachkräften.
- Differenzierte Finanzierungsmodelle nach Versorgungsstufen führen eine leistungsgerechte Vergütung von Krankenhausleistungen herbei.
- Bessere Ausbildungsanreize erhöhen die Attraktivität von Gesundheitsfachberufen und helfen, den Fachkräftemangel abzumildern.
- Verkürzte Anerkennungszeiten für ausländische Fachkräfte bewirken einen Defizitausgleich für den heimischen Fachkräftemangel.
- Ein Ausbau der Digitalisierung aller dafür im Krankenhaus geeigneten Arbeitsabläufe verbessert die Arbeitsprozesse, schont die knappen Personalressourcen und erhöht die Wirtschaftlichkeit.
